# Skin conditions in persons living with HIV during hospital admission in the UK

**DOI:** 10.1093/skinhd/vzaf116

**Published:** 2026-02-10

**Authors:** Jamie McCluskey, Louis Hickling, Collins Iwuji, David J Chandler

**Affiliations:** Department of Global Health and Infection, Brighton and Sussex Medical School, University of Sussex, Brighton, UK; Department of Global Health and Infection, Brighton and Sussex Medical School, University of Sussex, Brighton, UK; Department of Global Health and Infection, Brighton and Sussex Medical School, University of Sussex, Brighton, UK; The Lawson Unit, University Hospitals Sussex NHS Foundation Trust, Royal Sussex County Hospital, Brighton, UK; Department of Population Science, Africa Health Research Institute, KwaZulu-Natal, South Africa; Department of Global Health and Infection, Brighton and Sussex Medical School, University of Sussex, Brighton, UK; Dermatology Department, University Hospitals Sussex NHS Foundation Trust, Brighton General Hospital, Brighton, UK

## Abstract

**Background:**

The range of skin disease associated with advanced HIV infection is well described. Effective antiretroviral therapy has modified the spectrum of skin disease associated with HIV infection.

**Objectives:**

To characterize the profile of skin conditions occurring in a UK population of people living with HIV (PLHIV) during admission to a tertiary hospital.

**Methods:**

A retrospective review of inpatient admission records was conducted for all patients admitted to a tertiary hospital between January 2018 and December 2022. Data were collected using a standardized data extraction form and analysed with Microsoft Excel using descriptive statistics.

**Results:**

We identified 199 patients [median age 53 years; interquartile range (IQR) 41.5–63.0]. The median duration of HIV infection was 12.75 years (IQR 6.10–19.58), with a median duration on antiretroviral therapy of 8 years (IQR 0–14) and a median CD4 count of 368 (IQR 90–656) on admission. In total, 303 cases of skin disease were identified; the majority were skin infections (*n* = 164; 54.1%), followed by inflammatory dermatoses (*n* = 53; 17.5%), skin tumours (*n* = 39; 12.9%) and drug reactions (*n* = 13; 4.3%). In 11.2% of cases (*n* = 34), the diagnosis was unknown. Of the primary skin infections seen, 45.7% (*n* = 75/164) were bacterial, 26.8% (*n* = 44/164) were viral, 22.0% (*n* = 36/164) were fungal and 2.4% (*n* = 4/164) were parasitic. The most common condition was cellulitis, which occurred in 45 patients (22.6%). Sixty patients (30.2%) presented with advanced HIV infection (CD4 <200 cells mm^–3^). The most common conditions in this group were oral candidiasis (*n* = 21; 35%), herpes simplex virus infection (*n* = 12; 20%), seborrhoeic dermatitis (*n* = 9; 15%) and Kaposi sarcoma (*n* = 9; 15%).

**Conclusions:**

Cutaneous infection was the most common category of skin problem in this population of PLHIV during hospital admission. Bacterial cellulitis was the most common dermatological condition overall, while oral candidiasis was the most common condition in those with advanced HIV infection. Further work is needed to better characterize the profile of skin disease in PLHIV, including in larger prospective cohorts and outpatient settings.

## Teaser

Effective antiretroviral therapy has modified the skin manifestations of HIV. The aim of this study was to characterize the profile of skin disease in a UK-based population of people living with HIV (PLHIV). Of the 306 skin conditions identified, the majority were skin infections (54.6%), followed by inflammatory dermatoses (17.3%), skin tumours (12.7%) and drug reactions (4.2%). Cutaneous infection was the most common skin problem in this population of PLHIV during hospital admission. Bacterial cellulitis was the most common dermatological condition overall, whilst oral candidiasis was the most common condition in those with advanced HIV. Further work is needed to characterize the profile of skin disease in a larger population of PLHIV, including in an outpatient setting.

What is already known about this topic?

- The association between HIV disease stage and skin conditions has been well described.
- The introduction and widespread adoption of effective antiretroviral therapy has changed the spectrum of skin conditions seen in people living with HIV (PLHIV).

What does this study add?

- An up-to-date UK-based review of skin conditions in PLHIV admitted to hospital.
- Skin infections account for the majority of dermatological conditions occurring in PLHIV during hospital admission.
- Cellulitis was the most common condition.
- Despite the widespread availability of antiretroviral therapy in the UK, a significant proportion of this study population had advanced HIV infection (over half of whom were newly diagnosed with HIV during the admission).

Dermatological conditions are a well-recognized complication of HIV infection, with a lifetime risk of >90% of developing at least one skin condition.^1^ People living with HIV (PLHIV) are estimated to require over 15 times more visits to a dermatologist than the general population, representing a significant burden of cost of treatment and increased demand on dermatology services.^2^ In an untreated disease course, progressive immunocompromise leads to several HIV-associated dermatological conditions ranging from herpes zoster in early HIV, to Kaposi sarcoma and cryptococcal infection in later disease. With strong links between such diseases and the patient’s immune status, diagnosis of these conditions represents an opportunity to stage and predict progression of HIV disease.^3^

With the advent and widespread adoption of effective antiretroviral therapy (ART) the clinical manifestations of HIV and degree of immunocompromise in these patients has changed dramatically, reducing the frequency of dermatological diseases associated with advanced HIV infection.^4^ ART itself has also been implicated in several dermatological presentations such as adverse drug reactions (ADRs) and immune reconstitution inflammatory syndrome (IRIS)-related dermatosis.^5^ While some differences in the profile of skin disease in PLHIV reflect HIV disease stage, other variations can occur as a result of environmental, cultural or genetic differences.^6^

The aim of this study was to characterize the profile of skin disease in PLHIV admitted to a UK tertiary hospital. The secondary objective was to explore the possible association between the development and type of skin conditions and CD4 count in this population.

## Materials and methods

### Data collection

Data were extracted from the electronic inpatient records for all PLHIV admitted to a tertiary hospital between January 2018 and December 2022, and entered into a standardized data collection form in Microsoft Excel. This inpatient record was cross-referenced with long-term clinical data and HIV health monitoring data collected during presentation to the HIV outpatient department. Dermatological diagnosis was made based on clinical assessment by a medical doctor (­nondermatologist) during admission. Further laboratory investigations were requested to confirm certain diagnoses, such as viral polymerase chain reaction (PCR) testing on suspicion of mpox infection. Patients were eligible for inclusion if they were >18 years of age with confirmed HIV infection and had been diagnosed with a skin condition during hospital admission.

### Outcomes measured

The primary outcome measured in this study was the frequency of skin conditions diagnosed among the study participants. These skin conditions were categorized into skin infection (including primary skin infection and systemic infection with skin involvement) or were categorized as inflammatory dermatoses, malignancy, ADRs or unknown.

### Subgroups

Patients were separated into two subgroups based on immune status. Group 1 included individuals with well-controlled HIV (CD4 >200 cells mm^–3^), while group 2 comprised patients with advanced HIV infection (CD4 <200 cells mm^–3^). To achieve the secondary objective, the most frequent dermatological conditions observed between group 1 and group 2 were compared. Baseline demographic and clinical data were collected for all patients when available. This included sex, age, duration of HIV infection, CD4 count (cells mm^–3^), CD4:CD8 ratio and viral load at diagnosis, as well as ART regime and duration of ART at presentation.

### Statistical analysis

Results were analysed using descriptive statistics. A secondary statistical analysis comparing the prevalence of conditions between group 1 and group 2 was calculated using odds ratio (OR), 95% confidence interval (CI) and statistical significance set at a *P*-value of <0.05.

## Results

### Cohort characteristics

In total, 199 patients were included in the study, of whom 151 (75.9%) were men, 46 (23.2%) were women and 2 (0.9%) were trans-women. Median age was 53 years [interquartile range (IQR) 41.5–63.0]. Among all patients, the median duration of HIV infection was 12.8 years (IQR 6.1–19.6), with a median duration on ART of 8 years (IQR 0–14). Forty-six patients were ART naïve having been diagnosed with HIV on admission. The median CD4 count at presentation was 368 cells mm^–3^ (IQR 90–656) and the median viral load was 40 copies mL^–1^.

### Description of skin conditions

In total, 199 patients were diagnosed with 303 skin conditions (Table 1): 118 patients with a single dermatological condition, 58 patients with two conditions, 22 patients with three conditions and 1 patient with four conditions.

**Table 1:** Summary of skin conditions (*n* = 303) diagnosed in 199 participants

Category of skin condition	Skin infection	Inflammatory dermatoses	Malignancy	Drug reaction	Unknown
Number of cases (%)	164 (54.1)	53 (17.5)	39 (12.9)	13 (4.3)	34 (11.2)
Age (years), median (IQR)	52 (41–62)	52 (42–64)	59 (49–72.5)	50.5 (39–55.5)	52 (38–63)
Male sex	123 (75.2)	37 (69.9)	39 (87.2)	10 (76.9)	30 (88.2)
Duration of HIV infection (years), median (IQR)	12.2 (2.2–18.6)	15.3 (5.9–20.3)	15.0 (8.4–23.9)	9.8 (0–19.9)	14.3 (2.7–19.8)
Duration on ART (years), median (IQR)	8 (0–14)	9 (1–14)	7 (2–15)	8 (0–14)	9 (0–16.5)
CD4 (cells mm–3) on admission, median (IQR)	247 (46–662)	369 (197–742)	413 (170–690)	317 (216–624)	260 (52–587)
Advanced HIV infection (CD4 <200)	54 (32.9)	11 (20.8)	10 (25.6)	2 (15.4)	11 (32.4)
Viral load (copies mL–1), median (IQR)	1 337 763 (28 400–450 000)	48 947 (13 700–155 064)	63 300 (6450–250 000)	332 419 (13 800–1 416 120)	33 071 (1000–179 215)

ART, antiretroviral therapy; IQR, interquartile range.

Infection was the most common category of skin condition, representing 164 (54.6%) of total cases identified. Twenty-three different infections were identified, listed in order of frequency: cellulitis (*n* = 45; 27.4%), candidiasis (*n* = 28; 17.4%), herpes simplex virus (HSV) (*n* = 19; 11.6%), herpes zoster (*n* = 11; 6.7%), viral warts (*n* = 8; 4.9%), bacterial abscess (*n* = 7; 4.3%), dermatophytosis (*n* = 6; 3.7%), bacterial lesions (*n* = 5; 3.1%), syphilis (*n* = 5; 3.1%), systemic bacterial infection with skin involvement (*n* = 5; 3.1%), lymphogranuloma venereum (*n* = 4; 2.4%), mpox (*n* = 4; 2.4%), scabies (*n* = 4; 2.4%), bacterial folliculitis (*n* = 2; 1.2%), impetigo (*n* = 2; 1.2%) and necrotizing fasciitis (*n* = 2; 1.2%). The remaining infections were single cases of erysipelas, facial molluscum, *Malassezia* folliculitis, onychomycosis, herpetic lesion, herpetic whitlow and rectal chlamydia with skin involvement.

Inflammatory dermatoses were the second most common category of skin condition, representing 53 (17.5%) of the 303 skin conditions seen. Fifteen different inflammatory dermatoses were identified: seborrhoeic dermatitis (*n* = 17; 32%), eczema (*n* = 10; 19%), psoriasis (*n* = 9; 17%), dermatitis (*n* = 3; 6%), balanitis (*n* = 2; 4%), systemic lupus erythematous rash (*n* = 2; 4%) and vitiligo (*n* = 2; 4%). The remaining inflammatory dermatoses were single cases of hidradenitis suppurativa, IRIS-related rash, lichen planus, lichen sclerosis, localized lipoatrophy, oral hairy leucoplakia, cutaneous graft-versus-host disease and leucocytoclastic vasculitis.

Malignancy was the third most common category of skin condition, representing 12.9% (*n* = 39/303) of skin conditions seen. Seven malignancies were identified. The most common was Kaposi sarcoma (*n* = 21; 54%), followed by basal cell carcinoma (*n* = 9; 23%), squamous cell carcinoma (*n* = 3; 8%), melanoma (*n* = 2; 5%) and squamous cell carcinoma *in situ* (*n* = 2; 5%). The remaining were single cases of Merkel cell carcinoma and sarcoma, respectively.

Thirty-four (11.2%) of the 303 conditions did not have a confirmed diagnosis, consisting of undiagnosed rashes in 16 (47.1%), unidentified lesions in 9 (26.5%), unidentified ulcer in 6 (17.6%), fungating anal mass in 2 (5.9%) and subcutaneous nodule in 1 (2.9%). ADRs represented a smaller percentage of skin conditions seen, being responsible for 13 of 303 (4.3%).

Overall, the 5 most common conditions identified in this study were cellulitis (*n* = 45/303; 14.9%), candidiasis (*n* = 28/303; 9.2%), Kaposi sarcoma (*n* = 21/303; 6.9%), HSV virus (*n* = 19/303; 6.3%) and seborrhoeic dermatitis (*n* = 17/303; 5.6%). The largest group of skin conditions, infections, was further subcategorized by type of organism. Primary bacterial skin conditions were the most common group of infections, accounting for 75 (45.7%) of 164 infections seen. The next most common were primary viral (*n* = 44; 26.8%) and primary fungal (*n* = 36; 22.0%). Four (2.4%) infections seen were primary parasitic, and the remaining five (3.0%) were systemic bacterial infections with subsequent skin involvement.

### Advanced HIV infection

**Figure 1:**
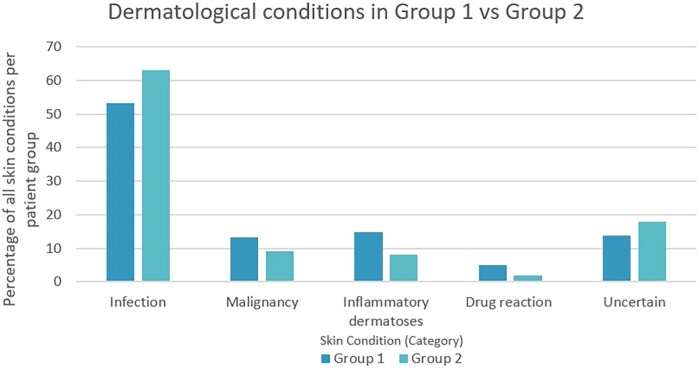
Percentage of dermatological conditions by category in group 1 (people with CD4 counts ≥200 cells mm^–3^) vs group 2 (people with advanced HIV).

Sixty patients (30.2%) presented with advanced HIV infection, characterized by a CD4 count <200 cells mm^–3^. The median age of patients included in group 2 was 52 years (IQR 39–60), with a median CD4 count of 30 cells mm^–3^ (IQR 9.5–104.0). The majority of this group (*n* = 32; 53.3%) were newly diagnosed with HIV on admission. One hundred conditions were diagnosed. The most common category of skin disease was also infection, accounting for 63.0% (*n* = 60) of the conditions identified. Skin infections were more common in group 2 than in group 1, accounting for 63.0%(*n* = 60) and 53.3% (*n* = 105) of all skin conditions, respectively. Figure 1 shows a comparison of the relative frequencies of the different categories of skin disease (as a percentage of all skin conditions).

**Figure 2:**
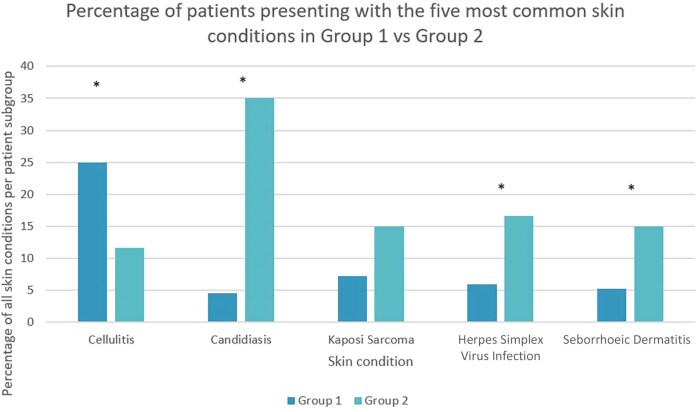
Percentage of patients presenting with the five most common dermatological conditions, separated by those with a CD4 count ≥200 cells mm^–3^ (group 1; *n* = 139) vs. advanced HIV infection (group 2; *n* = 60). Asterisks indicate significant differences between the two groups, *P* < 0.05.

Comparing the percentages of total cases of the five most common conditions overall in Figure 2, cellulitis was less common in group 2 (OR 0.40, 95% CI 0.17–0.95; *P* = 0.04), while candidiasis (OR 11.15, 95% CI 4.42–28.15; *P* < 0.001), HSV virus (OR 3.18, 95% CI 1.22–8.27; *P* = 0.02) and seborrhoeic dermatitis (OR 3.18, 95% CI 1.16–8.67; *P* = 0.02) were more common in group 2.

## Discussion

Skin infections occurring in PLHIV are diverse, accounting for considerable mortality, and have been reviewed recently.^7^ Bacterial cellulitis is an acute skin infection involving the dermis and subcutaneous tissues common in the general population.^8,9^ In this study, it was the most common dermatological condition, representing over one in five cases. One study analysing ambulatory visits found 100 cases/1000 of cellulitis in PLHIV,^10,11^ substantially higher than 32.5 cases/1000 in the general population. Risk factors for developing cellulitis in PLHIV include lower CD4 counts, higher HIV RNA levels and nonadherence to ART.^11^ This contrasts with our data and with other studies where the frequency of cellulitis was higher in those with CD4 counts ≥200 cells mm^–3^ and high adherence to ART.^12^

Candidiasis was the second most common condition identified overall and was present in over one-third of patients in group 2. The high prevalence of candidiasis in group 2 was expected, with the risk of developing candidiasis strongly linked to immunosuppression,^13^ particularly in those with CD4 counts ≤200 cells mm^–3^.^14^

The increased incidence of HSV in group 2 in this study is expected. HSV infection is well documented among PLHIV, with HSV-1 being almost universal and HSV-2 seroprevalence estimated between 50% and 95%.^15^ Reactivation of HSV is strongly associated with systemic physical stress, fever and microbial coinfection, all of which may be more prevalent in individuals with advanced HIV infection.^16^ Notably, previous studies have shown that HSV reactivations can persist even in minimally symptomatic PLHIV with robust CD4 counts, and may not be fully suppressed by ART adherence.^17,18^ While these studies used HSV viral shedding rates to assess clinical impact, they found that the percentage of days with reported HSV lesions was reduced with ART.^17^

Kaposi sarcoma, an AIDS-defining illness caused by human herpesvirus 8, was the third most common diagnosis in this cohort, typically associated with low CD4 counts.^19^ Notably, several cases occurred in group 1. Although global Kaposi sarcoma incidence has declined with widespread ART and better viral suppression, emerging evidence shows an increasing number of people presenting with higher CD4 counts.^20^ Seborrhoeic dermatitis is a frequent dermatological condition in PLHIV, with an incidence of up to 80%.^21^ While seborrhoeic dermatitis can occur at any stage of HIV infection, it often begins when CD4 counts are <450 cells mm^–3^ and increases in severity at <100 CD4 cells mm^–3^. The results of this study continue to support this.^21^

Forty-three of 199 patients (21.6%) in this study were newly diagnosed with HIV on admission; of these patients, 32 (74%) were newly diagnosed with advanced HIV infection. Data on HIV testing in the UK showed that 45% of new diagnoses in England in 2011 were made at a late stage.^22^ This higher rate of diagnoses of advanced HIV infection in our study may be reflective of the fact that the study population is made up of an inpatient population, likely with a deterioration in health that has required hospitalization and prompted HIV testing, compared with an outpatient population in early stages of the disease who are more likely to be asymptomatic and diagnosed on routine testing.

There remains a notable lack of UK data on dermatological conditions in PLHIV. The most significant prior work is a 1997 prospective study by Uthayakumar *et al*., conducted in Brighton, which examined 151 patients – 146 of whom were male – with a median age of 38.3 years, slightly younger than our cohort.^23^ All participants were assessed by an experienced dermatologist, leading to the identification of 331 skin conditions. The proportion of patients with advanced HIV infection (24.5%) was comparable to our study (30.2%). In both cohorts, infectious dermatoses were the most common, followed by inflammatory conditions. Interestingly, no cases of cellulitis were reported in the study by Uthayakumar et al.,^23^, probably reflecting its outpatient setting. However, ‘folliculitis and furunculosis’ were significantly more frequent among patients with CD4 counts <200 cells mm^–3^, potentially acting as precursors to cellulitis and subsequent bacteraemia. Dermatophyte infections of the skin and nails were more prevalent in their cohort (54%) – probably due to prospective recruitment and specialist dermatological assessment, explaining the greater number of overall diagnoses (331 conditions in 151 patients) vs. ours (303 conditions in 199 patients). This also helps account for the broader categorization in our cohort, with diagnoses such as bacterial lesions, herpetic lesions, bacterial ulcers and dermatitis being less specific.

International comparisons further contextualize our findings. Tan *et al.* conducted a similarly structured retrospective study in Lisbon, analysing records from 534 PLHIV attending their first consultation at a dermatology-focused HIV clinic.^24^ Our study had a higher proportion of patients with advanced HIV (30.2% vs. 17%). The pattern of skin disease differed markedly; anogenital human papillomavirus (HSV) was the most common diagnosis in the Lisbon cohort, followed by seborrhoeic dermatitis and nongenital HPV warts. In contrast, only five cases of HPV were recorded in our cohort, which may reflect differences in clinician expertise and examination thoroughness. Inpatient settings, where our data were collected, often involve less intimate examinations, potentially missing anogenital findings routinely identified in specialist outpatient clinics.

Halder *et al*. provide another point of comparison with a prospective study of the first 50 ART-naïve patients over the age of 14 years presenting to a dermatology outpatient clinic in India.^25^ Pruritic papular eruption (PPE) was the most frequently diagnosed condition (28%), with 96% of diagnoses confirmed histopathologically, ensuring high diagnostic accuracy. Our study, by contrast, identified no cases of PPE. This discrepancy may result from misdiagnosis by nonspecialist clinicians, with conditions like folliculitis or dermatitis potentially mistaken for PPE. Additionally, the higher prevalence of PPE in low-resource settings may account for the geographical variation between the Indian and UK cohorts.

Finally, it is crucial to consider the evolution of HIV care and dermatological diagnostics over recent decades. Advances such as improved access to viral PCR testing have enhanced the speed and precision of diagnosing cutaneous infections, while growing awareness among healthcare professionals of atypical dermatological presentations in PLHIV has ­probably facilitated earlier and more accurate identification of skin conditions.

This study has several important limitations. As a retrospective analysis, it is unclear whether comprehensive skin examinations were performed, raising the possibility that dermatological conditions were underdiagnosed or missed. Additionally, diagnoses were made by nonspecialists, which may have affected diagnostic accuracy, particularly for less common or atypical presentations. A further limitation is the absence of a comparator group of inpatients who did not have HIV under similar conditions, which restricts the ability to assess whether the patterns observed are specific to PLHIV or reflect broader inpatient trends.

Collectively, cutaneous infections are the most common skin conditions occurring in PLHIV during hospital admission. Of the cutaneous infections identified in this study, primary bacterial infections (predominantly cellulitis) accounted for most cases. In patients with advanced HIV, primary fungal infections (predominantly candidiasis) were most common. Also of note was the rate of Kaposi sarcoma in this cohort, as the third most common condition overall, including several unexpected cases documented in patients with robust CD4 counts. Greater than 10% of skin conditions seen in this study were unidentified, highlighting the need for senior dermatological input in PLHIV presenting with skin conditions. Further studies estimating rates of skin conditions in PLHIV may wish to consider early specialist input and microbiological confirmation to ensure accurate diagnosis. Finally, a significant number of patients were diagnosed with advanced HIV infection on admission, further emphasizing the need for regular HIV testing to ensure prompt diagnosis and initiation of treatment before patients develop advanced disease. Further research may seek to recruit patients prospectively over a longer period of time including in an outpatient setting.

## Author contributions

Jamie McCluskey (Conceptualization [supporting], Data curation [equal], Formal analysis [lead], Methodology [supporting], Project administration [supporting], Writing—original draft [lead], Writing—review & editing [equal]), Louis Hickling (Conceptualization [supporting], Data curation [equal], Formal analysis [equal], Investigation [equal], Methodology [supporting], Writing—original draft [supporting]), Collins Iwuji (Conceptualization [equal], Investigation [equal], Methodology [equal], Project administration [lead], Supervision [equal], Writing—review & editing [equal]) and David Chandler (Conceptualization [equal], Methodology [equal], Supervision [equal], Writing—review & editing [equal])

## Data Availability

The data underlying this article will be shared on reasonable request to the corresponding author.
